# Individuals on alert: digital epidemiology and the individualization of surveillance

**DOI:** 10.1186/s40504-018-0076-z

**Published:** 2018-06-14

**Authors:** Silja Samerski

**Affiliations:** Department of Anthropology and Cultural Studies, University of Bremen, Enrique-Schmidt-Str. 7, 28359 Bremen, Germany

**Keywords:** Quantified self medicine, Surveillance, Social and cultural implications, Securitization of the body, Generalization of suspicion

## Abstract

This article examines how digital epidemiology and eHealth coalesce into a powerful health surveillance system that fundamentally changes present notions of body and health. In the age of Big Data and Quantified Self, the conceptual and practical distinctions between individual and population body, personal and public health, surveillance and health care are diminishing. Expanding on Armstrong’s concept of “surveillance medicine” to “quantified self medicine” and drawing on my own research on the symbolic power of statistical constructs in medical encounters, this article explores the impact of digital health surveillance on people’s perceptions, actions and subjectivities. It discusses the epistemic confusions and paradoxes produced by a health care system that increasingly treats patients as risk profiles and prompts them to do the same, namely to perceive and manage themselves as a bundle of health and security risks. Since these risks are necessarily constructed in reference to epidemiological data that postulate a statistical gaze, they also construct or make-up disembodied “individuals on alert”.

## Introduction

Digital epidemiology promises exciting new insights into the occurrence and spread of diseases, into human behavior, into personal characteristics, and into the correlations among them. Its main goal is to detect health or security threats worldwide, in real time, rooted in the mining of online data, including personal data from social media and even information on health behaviors and health attitudes. In contrast to classical epidemiology that relied on reports from health experts, digital epidemiology draws on data that have been self-produced and usually for other purposes. The innumerable traces that people leave from their myriad activities online—from searching for information to Facebook posts—comprise its raw material. With a large proportion of the worldwide population leaving daily data traces of personal information, the new possibilities for health surveillance and control seem unbounded (Salathé et al. 2012).

Digitalized health surveillance not only facilitates the global monitoring of populations and security threats, but also the local monitoring of individual bodies and health risks. Digital technologies not only bring forth digital epidemiology and syndromic surveillance, but also eHealth, that is, personal health management on the basis of statistical analyses of individual data. Citizens sequence their DNA, routinely track their personal health and fitness status, subscribe to flu and other medical alerts, and manage themselves with health apps. With a new generation of sensors and trackers in the pipeline, this kind of individual surveillance is about to increase in scope, intensity and significance. Smart clothes, smart cars and smart houses are already on the market, and a “smart, ‘always-on’ health-monitoring system” that enables an “intimate, complete, non-invasive integration with people” is being developed (NSF 2017). A major aim of eHealth is to merge all health relevant data, be they self-tracked or medical, into a personal health file — an electronic health record — which then serves as the basis for personal and professional health decisions. In many countries, the electronic health record is already in use or about to be established. Highly digitalized societies such as Estonia have even gone a step further: “eEstonia” currently opens its digitalized health system for Big Data and plans to expand it into an “Health Information System 2.0” which is intended to work with the personal data of patients in real time (Grätzel 2016). Correspondingly, bioethicists debate the “moral obligation” of patients to release their data for data collections and analyses (Goodman 2010, 62). Yet, not only is the release of personal data about to become a new responsibility, but so also is data driven personal health management. As governmentality studies on health care have shown, patients are increasingly held responsible for their health by offering them tests and information on their risk profile (Weir 2006, Samerski 2015).

At first sight these developments seem to usher in a new era in medicine, namely “personalized” or “individualized medicine”, which eventually focuses on individuals instead of groups. Yet, all these personal data initially generated for individual purposes can only be interpreted in the light of epidemiology. Even the most personalized data double still needs reference to population statistics to be more than a database of random information. In order to “make sense” of a data double, be it for diagnosis, prevention or treatment, it has to be statistically analyzed, submitted to statistical classifications and correlations. This holds true for any statistical treatment of patients, be it in the realm of risk prevention or evidence based medicine. Yet, with advances in Big Data and digital epidemiology, this statistical analysis happens instantaneously and continuously. When patients’ data doubles are integrated into information systems, be they commercial or public, information and data flow in both directions: Patients’ data facilitate ever more refined predictive analytics, the core of Big Data applications, and the new statistical patterns and probabilities on possible future events in turn lead to new classifications of and risk imputations on patients’ data doubles. As Lupton and others have stated in the case of mHealth, digitalized data doubles “have a liveliness and vitality, a social life of their own that is facilitated by the app” (Lupton 2014, 615).

Yet, the direct submission of patients’ data doubles to continuous statistical analyses is only one side of the convergence. On the other side, digital health surveillance systems gain ever-increasing granular resolution, refine their scope from populations down to subgroups and finally to individuals. So far, global health surveillance focused on public and global health and was mainly implemented and used by health experts such as the military, public health departments, and the WHO (Velasco et al. 2014, Weir 2015). Systems like Google Flu Trends, too, did not make any statements about individual risks, but predicted the geographical and time referenced spread of an infection. Yet, with the securitization of public and global health, digital health surveillance increasingly targets individuals and their health management. Social scientists such as Lorna Weir and others have shown that public and global health has been securitized, meaning that the distinction between health as a social and humanitarian goal on the one hand and security as a political and military goal on the other hand has been blurred: today, if categorized as a security problem, health threats can provoke military interventions – as in the case of Ebola. Furthermore, Big Data and predictive analytics have broadened the scope of surveillance in general: “Now bulk data are obtained and data are aggregated from different sources before determining the full range of their actual and potential uses and mobilizing algorithms and analytics not only to understand a past sequence of events but also to predict and intervene before behaviors, events, and processes are set in train” (Lyon 2014, 4). This focus on the future, on the anticipation and prevention of possible events also reflects a conceptual transformation: with the help of digital epidemiology, the attention of syndromic surveillance has been shifted from professionally asserted health threats to events that create the potential for possible disease. This shift widens the space to establish what counts as relevant to health and security, including individual behavior (Weir 2015). For example, vaccination refusal can be interpreted as such potentially relevant event – in fact, digital epidemiology has already analyzed vaccination critics’ “sentiments” communicated in social media (Salathé and Khandelwal 2011). These developments show that self-surveillance and global surveillance, individual and population converge, both technically and epistemically. It is not new that epidemiology invites people to see themselves in the mirror of statistical laws and populations. Yet, with digitalization, the science of epidemiology gets ever more “individualized” while through digital media addresses users directly in a very personalized way. Thus, digital epidemiology leaks into people’s everyday lives with a new intensity and can have a profound impact on self-perceptions and social relations.

So far, the social and cultural implications of digital health surveillance have not become the subject of broad scholarly discussions. Yet, there is a small and growing shelf of insightful studies that have begun to investigate the social transformations instigated by individualized heath surveillance (a.o. Bauer and Olsén 2009, Cakici and Sanchez 2014, French 2009, Lupton 2014, Monahan and Wall 2007, Weir and Mykhalovsky 2010). Within this literature, however, the impact of surveillance, Big Data and feedbacks to individuals have not yet been analyzed in detail. Most studies investigate either surveillance through the rather sociopolitical lens of surveillance studies or eHealth in the field of the sociology of health. The close interconnectedness and convergence of digital epidemiology and eHealth, however, are mostly overlooked.

In this article, I show how individual and public health monitoring coalesce into a comprehensive health surveillance system which creates both a “world on alert” (Weir and Mykhalowskiy 2010) and “individuals on alert”. In order to do so, I will first expand and update David Armstrong’s analysis of “surveillance medicine”. As early as 1995, Armstrong argued that medicine organized around the concept of risk breaks with the key concepts and approaches of clinical medicine that traditionally was centered around the individual patient’s body. It is this break and the advance of surveillance medicine that paves the way for today’s melding of digital epidemiology with personal health care and the advance of “quantified self medicine”. In the second section, drawing on my research on the popularization of statistical constructs, I highlight three major impacts of surveillance medicine that have not yet been adequately discussed in the context of digital epidemiology: First, the epistemic confusion between statistical constructs and concrete statements about a person’s health; second, the fixation on probabilities that prompt people to live “on alert”, that is, in an “modus irrealis”, in an unreal mode; and third, the generalization of suspicion and its embodiment through popular scientific images of the body as a security apparatus.

## From surveillance medicine to quantified self medicine

People tracking themselves with various sensors and getting continuous digital feedback on their health performance is a powerful vision of scientists and entrepreneurs (Kraft 2017). When Armstrong analyzed surveillance medicine in 1995, this kind of digital self-surveillance was not yet on the horizon. However, his analysis is still relevant for today, since medicine today continues to focus on surveillance and risk. In contrast to surveillance medicine, with digital devices such as the smartphone and wearables, people are not so much controlled by experts, but rather quantify and control themselves. As Andreas Bernard has convincingly argued, within a few years techniques such as profiling or tracking systems, that were before exclusively used in the field of policing and crime control have now become attractive techniques for self-manifestation and empowerment (Bernard 2017). Thus, taking into account the recent rise of eHealth, I want to argue for the transformation from surveillance medicine to quantified self medicine. Since surveillance and risk also remain central concepts of today’s quantified self medicine, I will start with the immigration of “risk” into medicine and the shift from clinical medicine to surveillance medicine.

Traditionally, epidemiology and the healing arts have been two interrelated, but clearly distinguished fields. Epidemiology addressed populations whereas traditional clinical medicine addressed individuals. Yet, since the 1960s, epidemiology advanced to a guiding discipline in medicine, slowly changing the concepts and practices of the latter. Only at the beginning of the twentieth century did the term “risk” emigrate from insurance and business into colloquial German where it gained acceptance as a synonym for “danger” and “daring”. In 1934 there was talk about the risks inherent in street traffic, and in the 1960s health apostles promoted filter cigarettes as “risk-free tobacco products”. It is only in the 2nd half of the twentieth century that the “risk factor model” not only explained the distribution of diseases in populations, but also started to guide medical theory and practice. It is only some decades since doctors impute personal risks to their patients, an alleged prognosis, which then hangs over their present-day lives like a Damoclean sword (Armstrong 1995, Samerski 2015). Thus, the concept of risk can be understood as the interface between the two strategies of biopower, the disciplinary and the regulatory. Michel Foucault poignantly describes the rationality of epidemiology as a regulatory strategy that aims at establishing a homeostasis of statistical – or epidemiological - phenomena, such as birth rates, mortality, morbidity, etc. It is “a technology which brings together the mass effects characteristic of a population, which tries to control the series of random events that can occur in a living mass, a technology which tries to predict the probability of those events (by modifying it, if necessary), or at least to compensate for their effects. This is a technology which aims to establish a sort of homeostasis, not by training individuals, but by achieving an overall equilibrium that protects the security of the whole from internal dangers” (Foucault 2004, 249).

Today, doctors’ offices are filled with people robbed of their sense of well-being, not by an actual adversity but by risk predictions, that is the attempt to control probabilities. Whether pandemics, early aging, an exceptional child, or lumps in a breast—in the “risk society” (Beck 1992), everything that may happen is anticipated as a probability or risk. However, “risk” does not identify a concrete reality but only a specific form of objectifying potential events. Risks in themselves do not exist. Conversely, this means that everything can be made into a risk: “Nothing is a risk in itself. There is no risk in reality. But, on the other hand, anything *can* be a risk” (Ewald, 1991, 199). Thus, by threatening patients with potential future calamities, risk attestations urge them to adapt their health management to statistical laws and to the demands of security technologies.

The early statisticians of the nineteenth century were very aware of the heterogeneity between the regularity of the masses and the individual case, between the calculated and the concrete. The Belgian mathematician Quételet, the inventor of the “average man” (“l’homme moyen”), expressly warns against drawing conclusions about individuals based on statistical laws: he forcefully stated that these laws, in accordance with the manner of their determination, no longer have anything of a specific individual about them. No concrete individual was reflected by the mirror of the “average man”. Any application to an individual person would essentially be false; it would be like using a mortality table to determine when a certain person will die (cited in Ewald, 1993, 196). Yet, with the rise of a new governmental regime centered around “security”, as well as with refined statistical techniques and automated computational power, citizens have increasingly come to be treated as statistical cases, as faceless risk profiles. People are not governed in relation to their individuality or identity but as members of populations. The embodied individual is of interest to governments insofar as the individual can be identified, categorized and recognized as a member of population (Ruppert 2011, 158).

The rise of risk in medicine in the late twentieth century mirrors this predominance of regulatory techniques of biopower and the corresponding transformation of individuals to faceless risk profiles. No matter if pregnancy, cancer, stroke or simply a headache – whenever patients encounter a doctor today, they are likely to receive a risk assessment. “Calculating and recalculating risk profiles has become part of the core task of medicine” (Armstrong 2011, 158). As early as 1995, medical sociologist David Armstrong providently described how this risk-centered medicine differs fundamentally from the previous regime of clinical medicine. It does not emanate from the concrete body of the patient, but is derived from statistical collectives and probability spaces. In clinical medicine, the doctor would read and interpret symptoms, which indicated a hidden lesion or disease in the body. In contrast, in a risk centered medicine, the doctor detects risk factors that indicate a future threat, a statistically anticipated disease potential. Thus, risk in medicine blurs the difference between “normal” and “pathological,” which had hitherto shaped medical thinking and acting. Medical genetics, for instance, assigns mutated genes and genetic risks to healthy people and turns them into asymptomatic patients – not knowing if they will ever fall sick. Prenatal diagnostics declares that all pregnant women are in need of care – even if nothing is wrong with them. No longer are the ill the sole targets of medical monitoring and treatment. Instead, today’s medicine targets the healthy population in particular and “requires the dissolution of the distinct clinical categories of healthy and ill as it attempts to bring everyone within its network of visibility” (Armstrong 1995, 395). As early as 20 years ago, David Armstrong called this new trend in medicine “surveillance medicine”. Its remit and scope are boundless, because it aims at controlling tomorrow’s uncertainty: “Surveillance Medicine […] attempt[s] to transform the future by changing the health attitudes and health behaviours of the present” (Armstrong 1995, 402).

This transformation from clinical medicine to surveillance medicine has laid the grounds for quantified self medicine, that is the current amalgamation of population surveillance and self-surveillance. Today, with Big Data, digital epidemiology and eHealth, surveillance medicine is not only technically intensified, but also epistemically generalized. Bauer and Olsén describe the new digitalized monitoring techniques as “distributed surveillance” (2009, 126) that universalize the “population gaze” that is constitutional for the regulatory techniques of biopower. In the 1990s, when Armstrong’s analyzed surveillance medicine, the epidemiological knowledge was constructed by experts in corresponding institutions; the panoptical gaze was still restricted to experts. Today, however, through clinical monitoring, self-tracking and digital data flows, patients are not only objects but also subjects of data generation and reconstruction. They are not only disciplined by internalizing the fact of being the object of observation, but are also invited to occupy a panoptical perspective themselves — on their own body as well as on others. By tracking their health and interpreting their data in the light of population statistics, they become subjects and objects of surveillance and epidemiological analysis at the same time. They are envisaged to lead a “self-monitored life that navigates through the grids of potential health threats” (Bauer and Olsén 2009, 125). This “self-monitored life” in the light of statistical correlations and predictions, however, entraps individuals in paradoxes with far reaching consequences. What at first glance might look like a step towards empowerment — the loss of a professional and institutional monopoles on knowledge and surveillance — at second sight turns out to transform individuals into disembodied risk profiles; they become “individuals on alert” that adapt their life and health management to the threats and demands constructed by a global security regime.

## Epistemic confusions: “Personal risk”

By lending the appearance of personal significance to statistical constructs, the concept of risk, or rather, the oxymoron of “personal risk” works as the main glue for the agglutination of population surveillance and self-surveillance. Inevitably, data driven health surveillance oriented towards anticipation and prevention is based on the calculation of probabilities, no matter if an app monitors depression or syndromic surveillance monitors the flue. In the consulting room, these probabilities are boiled down into chances and risks, actuarial notions that are then understood by the client as threats. Health professionals, health apps and patients alike interpret risk factors as “objective clinical signs of disease” (Gifford 1986, 222), thereby evoking a new reality that Lorna Weir calls “clinical risk”. Clinical risk appears as something patients can “have”– just like a sore tooth or a stomach ulcer. Yet, by definition, probabilities quantify frequencies in populations but make no predictions about individual cases. Thus, “clinical risk comprises an unstable amalgam of incompatible forms of reasoning” (Weir 2006, 19).

This inherent paradox of risk in medicine is revealed when doctors are asked to explain what the risk assessments mean. As the following excerpt from a genetic counseling session shows,^1^ they immediately get entrapped in contradictions: a genetic counselor explains to her healthy client that **“**when a mutation in BRCA1 or 2 is carried, (…) then a woman who carries this mutation, from a statistical perspective, which says nothing at a personal level, has lifelong, an approximately 80-85 percent risk of developing breast cancer.” And a minute later she states that this number is “very high” and that the troubled women should “be careful” and choose regular checkups. How can a statistical imputation that “says nothing at a personal level” inform what a patient should do? Spelled out, the counselor has made the following statement about her client: If she had 100 lives, then in 80–85 of these lives she would develop breast cancer, and in 15-20 lives she would not. In reality, however, the woman has but her one life. What happens to her in this one life – the only question meaningful for her – is still written in the stars (Samerski 2015).

This class of statements – statistical speculations—quantifications of possible futures, will proliferate with digital health surveillance. As Lyon states, “Big Data fosters an anticipatory, future tense approach to surveillance” (Lyon 2014, 10), aiming “to predict and preempt future developments” (Lyon 2014, 10). The more data gathered, the more risks and predictions will be produced. All kinds of markers and behaviors, from genetic mutations to susceptible behavior or variables such as smoking, age, sex, etc. can be correlated with health outcomes (Jensen, Jensen and Brunak 2012). People might get feedbacks and alerts for an increasing number of health risks such as the flu, depression, smog, allergy, heart attack, obesity, high calorie uptake, gastroenteritis or rubella. And whereas the genetically counseled women are still aware that the risk they face from a supposed genetic mutation is the result of expert deductions, people in future are likely to confer a misplaced concreteness to the risk predictions they confront. The questionable procedures that generate a risk statistic – for example, a 60% chance of X — will remain invisible and unquestioned.

## Living in modus irrealis

Risk fixes the gaze on a possible future and paralyzes one’s sense of the present. People are being asked to be where they are not and perhaps never will be. Furthermore, in the context of health, risks turn imaginary possibility into latency. They conjure up a future calamity that seems to lurk in one’s own body. The “either-or” – it may happen or not – mutates into a “not yet.” The anticipated future, predicted by analogy to a game of chance, is reshaped as a concealed present. Thereby, despite good health, a risk assessment transforms the body into a source of latent harms. For this reason, a woman assessed with an elevated risk for ovarian cancer after a PAP test wants to have everything removed that is not necessary to her life: “Because the tiniest bit can go wrong, and if that’s not there, well, you can’t have a problem with it” (Kavanagh and Broom 1998, 440).

With predictive analytics being a main aim of digital epidemiology, the anticipation of speculative possibilities will become everyday routine. The main aim of Big Data is predictive analytics, that is the anticipation and prevention or at least manipulation of future events. In the same way that predictive policing is supposed to detect the criminal before he has committed the crime, so predictive medicine is to detect the risk carrier before the outbreak of disease. In grammar, the speculative mode of identifying what is imagined, speculative, and fanciful is called the irrealis mood, or subjunctive mood. The Brother Grimms’ tale of “Clever Elsie” tells how the anticipation of a speculative future generates a helpless paralysis in the present. Clever Elsie is a symbol for life in irrealis mood that, in the times of predictive analytics, is about to become everyday reality: Elsie remains sitting in the cellar crying and paralyzed under a walled-in pick-axe because this could kill her child who not yet born. Upstairs, Hans, her suitor, is waiting with her parents. Glancing at the pick-axe, Elsie anticipated their future together and a possible misfortune: If she marries Hans and has children, and if she one day sends her child down to the cellar for beer, then it could be killed by the falling pick-axe. Pondering this menace, Elsie remains sitting and lamenting this imagined fate.

Risk alerted people spend their lives in this artificial “not yet”. Since there are no tangible, present, and perceptible reasons for their anxiety, they cannot free themselves from this shadow. Risks that are made up by a tissue of probabilities which cannot be experienced or perceived, generate a free-floating and vague fear or sense of dread that cannot be pacified by reason. And with Big Data, the elaboration of risk profiles become ever more unintelligible. Therefore, surveillance medicine renders one helpless and generates a boundless need for reassurance: for further surveillance as well as for tests offering assurance that the evoked calamity — probably — is not yet there.

## The generalization of suspicion and its embodiment

The anthropologist Emily Martin has examined how social relationships are mirrored in our understanding and experience of the body (Martin 1987). In an unsettling way, social demands coincide with the scientifically transmitted view of the (female) body. Whereas industrial society conceived the body in terms as “production” and “hierarchy,” the post-industrial age of self-responsibility, flexibility, and self-management has increasingly led to a description of the body as a “flexible body” (Martin 1994), its epitome being the immune system. The immune system requires constant monitoring and optimizing – in the same way that modern workers must always manage and optimize themselves to satisfy the demands of the post-industrial economy.

In the twenty-first century, genetics is one of the sites where a body which dovetails with the social precepts of the time is constructed: a body in need of surveillance, a body that is constantly threatened from within because it contains unfathomable mutations and pre-programmed risks. A brief dialogue from cancer genetic counseling illustrates well how closely today’s popularized scientific body corresponds to the social ideologemes of security and surveillance. The geneticist says: “This is a so-called mismatch repair, which means it is a function by which, (…) when random mistakes occasionally happen, this corrects them. (…) There are specific protein molecules in the body that are in charge of this.” The client replies: “Like the police”. The geneticist immediately confirms: “Exactly, they are like the police. And this is ex…this is exactly the function of these genes. (…) And (…) when they do not function right, then logically such changes can persist. Other genes, (…) like the tumor suppressor genes, are on guard to make sure cells do not further mutate. And when they, when they become nonfunctional, then cancer can develop” (Samerski 2015, 103). Such criminological pictures are common in popular scientific representations. The Epigenome Network of Excellence, for example, has excessively used them in earlier versions of its website: “Cancer is the ‘enemy within’, the criminal element that upsets the harmony of our body’s cellular community. Our internal police force, our immune system, does all within its powers to hunt down and disarm these troublemaking cells”. (Samerski 2015, 103).

Apparently, the counseling client of the above extract has already been exposed to such criminological images and now pictures her own body as if it were a modern surveillance system; she sees herself as being under surveillance by a patrolling police force. The biggest enemy, these explanations suggest, does not invade from outside, but comes from inside and is part of one’s system. A biologist makes this analogy with anti-terrorist security explicit: Genes are like “terrorists”, the biologist John Turner writes: “They have the power to kill, maim, or make life downright miserable for us and our children. Some strike at birth, others ‘sleep’ for decades, and, like good terrorists, they are so well integrated into our body politic that, until the last few years, their exact whereabouts were a mystery: their individual extirpation (or more properly correction) is still well-nigh impossible” (Turner 2001, 8).

This securitization of personal health, the imputation of an unsafe, precarious body within which lurk imminent threats and ominous futures, is far-reaching. First, these threats are imperceptible. In order to feel secure, at least for a short moment, the inhabitants of such a body are in constant need for surveillance and control. They cannot trust their senses anymore and literally embody the need for self-surveillance and checkups. Second, everybody is turned into a suspect. In the field of predictive policing where basically the same techniques of statistical anticipation and surveillance are implemented, criminologists call this the “generalization of suspicion”. Everybody is seen as a potential thief or terrorist until proven otherwise. Thus, technologies of prevention precariously invert a legal concept that is fundamental for a constitutional democracy: The legal concept of innocent until proven guilty. After the assessment of an increased risk, the counseled women, young and healthy, will remain a cancer suspect until a test lowers her risk to average. Once diagnosed as “at risk”, and this happens to whole populations, people stay suspect until they manage prove their health or innocence.

## Conclusions

By analyzing digital epidemiology in the context of other surveillance techniques including eHealth and self-tracking, this article has broadened the scope of discussion: It has identified three social and cultural implications that come into view when the epistemological and practical links between population surveillance and self-surveillance are made visible. By inviting patients and users to adopt a statistical gaze on themselves, digital epidemiology contributes to a disturbing transformation of health, disease and body. With the avalanche of digital data that brings everyday practices and actions as well as sentiments and social relations into the field of visibility, the possibilities for generating and attesting to pathogenic risks are limitless. Furthermore, digital devices integrate people into surveillance systems, so that the statistical alerts and feedbacks directly inform personal orientations and actions. In this world of digital health surveillance, the doctor largely disappears. He might be reduced to an operator of “Watson health” and its counterparts, facilitating the interaction between patients and digital health technologies.

The term “health” once indicated a void, it meant unhurt, in sound condition. A healthy person did not miss anything. Today, striving for “health” saddles people with countless – and meaningless – risks and generates the need for surveillance. Perhaps Aldous Huxley saw most deeply into the contemporary situation when he said, “Medical science has made such tremendous progress that there is hardly a healthy human left.”

## Footnotes

1 I have described elsewhere the methodological details and ethics protocol governing the research use oft he human subjects’ data-set from which the following quotations are drawn. See Samerski 2002, Samerski 2015.
